# Deletion of microRNA-183-96-182 Cluster in Lymphocytes Suppresses Anti-DsDNA Autoantibody Production and IgG Deposition in the Kidneys in C57BL/6-Fas^lpr/lpr^ Mice

**DOI:** 10.3389/fgene.2022.840060

**Published:** 2022-07-07

**Authors:** Zhuang Wang, Bettina Heid, Ran Lu, Mohit Sachdeva, Michael R. Edwards, JingJing Ren, Thomas E. Cecere, Deena Khan, Taschua Jeboda, David G. Kirsch, Christopher M. Reilly, Rujuan Dai, S. Ansar Ahmed

**Affiliations:** ^1^ Department of Biomedical Sciences and Pathobiology, Virginia-Maryland College of Veterinary Medicine (VMCVM), Virginia Tech, Blacksburg, VA, United States; ^2^ Preclinical Lead Immunology, Spark Theraprutics, Philadelphia, PA, United States; ^3^ Department of Pharmacology and Cancer Biology, Duke University Medical Center, Durham, NC, United States; ^4^ Edward Via College of Osteopathic Medicine, Blacksburg, VA, United States

**Keywords:** epigenetics, miR-183-96-182 cluster, autoantibody, inflammatory cytokine, systemic lupus erythematosus, murine model

## Abstract

Dysregulated miRNAs have been implicated in the pathogenesis of systemic lupus erythematosus (SLE). Our previous study reported a substantial increase in three miRNAs located at the miR-183-96-182 cluster (miR-183C) in several autoimmune lupus-prone mice, including MRL/lpr and C57BL/6-lpr (B6/lpr). This study reports that *in vitro* inhibition of miR-182 alone or miR-183C by specific antagomirs in activated splenocytes from autoimmune-prone MRL/lpr and control MRL mice significantly reduced lupus-related inflammatory cytokines, interferon-gamma (IFNγ), and IL-6 production. To further characterize the role of miR-182 and miR-183C cluster *in vivo* in lupus-like disease and lymphocyte phenotypes, we used hCD2-iCre to generate B6/lpr mice with conditional deletion of miR-182 or miR-183C in CD2^+^ lymphocytes (miR-182^−/−^B6/lpr and miR-183C^−/-^B6/lpr). The miR-182^−/−^B6/lpr and miR-183C^−/−^B6/lpr mice had significantly reduced deposition of IgG immunocomplexes in the kidney when compared to their respective littermate controls, although there appeared to be no remarkable changes in renal pathology. Importantly, we observed a significant reduction of serum anti-dsDNA autoantibodies in miR-183C^−/−^B6/lpr mice after reaching 24 weeks-of age compared to age-matched miR-183C^fl/fl^B6/lpr controls. *In vitro* activated splenocytes from miR-182^−/−^B6/lpr mice and miR-183C^−/−^B6/lpr mice showed reduced ability to produce lupus-associated IFNγ. Forkhead box O1(Foxo1), a previously validated miR-183C miRNAs target, was increased in the splenic CD4^+^ cells of miR-182^−/−^B6/lpr and miR-183C^−/−^B6/lpr mice. Furthermore, *in vitro* inhibition of Foxo1 with siRNA in splenocytes from miR-182^−/−^B6/lpr and miR-183C^−/-^B6/lpr mice significantly increased IFNγ expression following anti-CD3/CD28 stimulation, suggesting that miR-182 and miR-183C miRNAs regulate the inflammatory IFNγ in splenocytes via targeting Foxo1. The deletion of either miR-182 alone or the whole miR-183C cluster, however, had no marked effect on the composition of T and B cell subsets in the spleens of B6/lpr mice. There were similar percentages of CD4^+^, CD8^+^, CD19^+^, as well as Tregs, follicular helper T (T_FH_), germinal center B (GCB), and plasma cells in the miR-183C^−/−^B6/lpr and miR-182^−/−^B6/lpr mice and their respective littermate controls, miR-183C^fl/fl^B6/lpr and miR-182^fl/fl^B6/lpr mice. Together, our data demonstrate a role of miR-183C in the regulation of anti-dsDNA autoantibody production *in vivo* in B6/lpr mice and the induction of IFNγ in *in vitro* activated splenocytes from B6/lpr mice.

## Introduction

Systemic lupus erythematosus (SLE) is a prototypical autoimmune disease characterized by autoantibodies against cellular components and systemic damage to multiple organs, especially kidneys. However, due to the complexity of this disease, the cause of SLE remains unclear. Various risk factors, including genetic, epigenetic, environmental, and hormonal factors, are believed to contribute to the pathogenesis of SLE (Ansar Ahmed et al., 1985; Squatrito et al., 2014; Parks et al., 2017; Goulielmos et al., 2018). Recently, there has been a significant focus on the epigenetic regulation of SLE, especially the role of microRNAs (miRNA), in lupus pathogenesis (Honarpisheh et al., 2018; Dai et al., 2021).

The evolutionally conserved miRNAs play crucial roles in various biological processes, including the development and function of the immune system (Baltimore et al., 2008; Baumjohann and Ansel, 2013; Danger et al., 2014; Mehta and Baltimore, 2016). Dysregulated miRNA expression and function have been identified and implicated in the pathogenesis of various human diseases. Signature lupus-related miRNAs have been implicated in disease pathogenesis by regulating the key molecules and signaling pathways involved in lupus, such as DNA methylation and type I interferon pathways, leading to abnormal production of inflammatory cytokines, chemokines, and antibodies (Tang et al., 2009; Pan et al., 2010; Dai and Ahmed, 2011; Shen et al., 2012; Zan et al., 2014; Dai and Ahmed, 2016; Schell and Rahman, 2021). In our previous miRNA profiling study with three genetically different murine lupus models (C57BL/6-lpr or B6/lpr, NZB/NZW_F1_, and MRL/lpr), we identified a common upregulation of a group of miRNAs, including three miRNAs from the highly conserved miR-183-96-182 cluster (miR-183C) (Dai et al., 2010). Notably, the upregulation of miR-182 and miR-183 of miR-183C has also been identified in human lupus in the recent studies (Chen J. Q. et al., 2017; Lin et al., 2020).

The miR-183 cluster was initially identified as a sensory organ-specific miRNA cluster (Xu et al., 2007). The high expression of miR-183C has been identified in mouse sensory organs such as the inner ear, eye, and olfactory bulb (Weston et al., 2006; Bak et al., 2008; Pierce et al., 2008; Li et al., 2010). Typically, the miR-183C expression is very low in the lymphoid tissues in resting state (Xu et al., 2007). However, miR-183C miRNAs are highly expressed in activated immune cells (Stittrich et al., 2010; Pucella et al., 2015; Li et al., 2016). Current studies have revealed that miR-183C miRNAs are critically involved in immunity and autoimmunity by regulating B cell functions, T cell expansion and activation, the differentiation and function of Tregs, and pathogenic Th17 cells (Stittrich et al., 2010; Ichiyama et al., 2016; Li et al., 2016; Wan et al., 2016; Ichiyama and Dong, 2019). While miR-182 knockout B6 mice have normal B cell and T cell development, these mice have impaired extrafollicular B cell antibody response (Li et al., 2016). *In vitro* studies have shown that miR-182 is highly induced by IL-2 to promote T helper (Th) cell expansion and proliferation by targeting Foxo1 (Stittrich et al., 2010). Specific inhibition of miR-182 in Th cells suppressed Th cell expansion and decreased ovalbumin (OVA)-induced arthritis in mice. A positive correlation between miR-182 expression and the severity of induced experimental autoimmune encephalomyelitis (EAE) was demonstrated in B6 mice (Wan et al., 2016). The authors demonstrated that during EAE development, miR-182 suppressed CD4^+^CD25^+^Foxp3^+^ Tregs differentiation by targeting Foxo1 (Wan et al., 2016). Another study reported that miR-183C cluster miRNAs were highly induced by IL-6-STAT3 signaling, which promoted the differentiation of pathogenic Th17 cell differentiation by targeting Foxo1 (Ichiyama et al., 2016). The deletion of miR-183C significantly reduced the severity of EAE disease by suppressing the pathogenic function of Th17 cells (Ichiyama et al., 2016).

In SLE, we have reported that the miR-183C miRNAs were highly upregulated in splenic lymphocytes in three different murine lupus models and correlated with the disease development (Dai et al., 2010). Additionally, Choi and coworkers reported highly upregulated miR-183C in another murine lupus model, the C3. MRL-Faslpr/J model, which further validates the association of miR-183C with murine lupus (Choi et al., 2016). The treatment of C3. MRL-Faslpr/J mice with cyclophosphamide or human-derived mesenchymal stem cells reduced not only classical lupus parameters (anti-dsDNA, immune complex C3 deposition, and CD138^+^ cells) but also the miR-182 and miR-96 expression (Choi et al., 2016). Inhibition of miR-182 *in vivo* in MRL/lpr mice with the antagomir-182 treatment resulted in the amelioration of lupus nephritis, which provided additional support for a potential role of miR-182 in lupus (Wang X. et al., 2018).

The miR-182 has been shown to be largely dispensable for adaptive immune cell development and function in B6 mice (Pucella et al., 2015). This may likely be due to the functional compensation by the other two miRNAs (miR-96 and miR-183) within the miR-183C since they have similar seed sequences and could target the same gene, such as Foxo1. On the other hand, the minor differences in the seed sequences will allow individual miR-183C miRNA specific unique targets (Dambal et al., 2015). Individual miR-183C miRNA may play a distinct role in different aspects of immune function. For example, miR-96, but not miR-182 and miR-183, was critical for the induction of pathogenic Th17 cytokines (Ichiyama et al., 2016), while miR-182, but not miR-96 and miR-183, was involved in IL-2 driven helper T cell expansion and function (Stittrich et al., 2010). Therefore, in this study, to further characterize the role of miR-183C and miR-182 in lupus *in vivo*, we derived both miR-183C^−/−^B6/lpr and miR-182^−/−^B6/lpr mice with conditional deletion of whole miR-183C or miR-182 alone in CD2^+^ lymphocytes. Here, we report the immunological and pathological consequences of *in vivo* deletion of miR-182 and miR-183C in B6/lpr mice.

## Material and Methods

### Mice

All animal experimental procedures and housing have been approved by the Institutional Animal Care and Use Committee (IACUC) of Virginia Polytechnic Institute and State University. In this study, MRL/lpr (MRL/MpJ-Fas^lpr^/J, Stock No. 000485), B6/lpr (B6. MRL-Fas^lpr^/J, Stock No. 000482), MRL (MRL/MpJ, Stock No.000486), B6 (C57BL/6J, Stock No. 000664) and hCD2-iCre (B6-Cg-Tg (CD2-icre)4Kio/J, Stock No. 008520) mice were purchased from Jackson Laboratory (JAX, Maine, United States) and bred in-house.

Although the MRL/lpr model has been widely used to study human lupus, it has several drawbacks that prevent it from being easy to generate MRL/lpr mice with specific gene deletion. This include that MRL/lpr mice have a mixed genetic background. Consequently, multiple generations of backcross are needed to generate knockout mice in MRL/lpr background. It is even more difficult and time-consuming for generating conditional knockout MRL/lpr mice since the commercially available CRE transgenic mouse stains are in B6 background. We, therefore, generated autoinflammatory-prone B6/lpr mice with conditional deletion of either miR-183C cluster or miR-182 in lymphocytes to investigate the role of miR-183C and miR-182 in lupus *in vivo*. We bred hCD2-iCre mice with B6/lpr mice in-house to generate CD2-CreB6/lpr mice. The miR-183C^fl/fl^ and miR-182^fl/fl^ mice in a B6-129/SvJ mixed genetic background were generated by Dr. David G. Kirsch’s group at Duke University Medical Center. The development and characterization of miR-182^fl/fl^ mice on a B6-129/SvJ mixed genetic background have been described in detail in a previous report (Sachdeva et al., 2014). An identical strategy was used to generate the miR-183C^fl/fl^ mice on a B6-129/SvJ mixed genetic background (Supplementary Figure S1) except that the loxP sites flank the entire miR-183, miR-96, miR-182 cluster instead of only miR-182. The miR-183C^fl/fl^ and miR-182^fl/fl^ mice in the mixed background were backcrossed with B6 mice for at least eight generations. The miR-183C^fl/fl^ and miR-182^fl/fl^ mice in a B6 background were crossbred with B6/lpr to generate miR183C^fl/fl^B6/lpr and miR-182^fl/fl^B6/lpr mice, which were then crossbred with CD2-CreB6/lpr mice to generate CD2-CremiR-183C^fl/fl^B6/lpr (miR183C^−/−^B6/lpr) and CD2-CremiR-182^fl/fl^B6/lpr (miR-182^−/−^B6/lpr) mice. The miR183C^−/−^B6/lpr and miR-182^−/−^B6/lpr mice were bred with miR-183C^fl/fl^B6/lpr and miR-182^fl/fl^B6/lpr mice, respectively to obtain experimental miR183C^−/−^B6/lpr and miR-182^−/−^B6/lpr mice. The littermate miR183C^fl/fl^B6/lpr and miR-182^fl/fl^B6/lpr, which do not carry the hCD2-iCre gene, served as wild-type control B6/lpr mice for conditional miR183 and miR-182 knockout B6/lpr mice. Due to the female predominance of lupus, only female mice were used in this study.

All mice were housed in our Association for Assessment and Accreditation of Laboratory Animal Care **(**AAALAC)-certified animal facility at the Virginia-Maryland College of Veterinary Medicine (VMCVM), Virginia Tech. Mice were fed with a commercial 7013 NIH-31 Modified 6% Mouse/Rat Sterilizable Diet (Harlan Laboratory, Madison, WI, United States) and given water *ad libitum*. The mice at designated age were euthanized by CO_2_ asphyxiation according to the IACUC approved protocol.

### Measurement of Proteinuria

Proteinuria was measured by dipstick analysis using Chemistrip-2GP (Roche Diagnostics Corporation, Indianapolis, IN, United States). The semi-quantitative scale was demonstrated as follows: “-”: negative or trace; “+” 30 mg/dl; “++”: 100 mg/dl and “+++”: ≥500 mg/dl.

### Blood Collection and Serum Preparation

Peripheral blood was collected every 4 weeks by retro-orbital bleeding following IACUC requirements. Freshly-collected blood was maintained in 4°C refrigerator overnight to clot, and then centrifuged at 1,500× g for 15 min at 4°C to remove clots and collect supernatant as serum. The serum was stored at −80°C freezer.

### Splenic Lymphocyte Preparation, Splenic T and B Cell Purification, and Cell Culture

Whole splenocytes were prepared by following standard laboratory procedures described in detail previously (Lengi et al., 2007; Edwards et al., 2017; Dai et al., 2019; Edwards et al., 2020). Per the manufacturer’s protocol, splenic CD4^+^ cells (T cells) and CD19^+^ cells (B cells) were purified from splenic lymphocytes with anti-CD4 (L3T4) and anti-CD19 MicroBeads (Miltenyi Biotec, San Diego, CA, United States), respectively. The purity of CD4^+^ (93.84% ± 2.03%) and CD19^+^ (91.88% ± 1.81%) cells were confirmed by flow cytometry.

To activate splenic lymphocytes, 2.5 × 10^6^ cells were seeded in 24-well plates and stimulated with 500 ng/ml of Lipopolysaccharide (LPS, from Sigma-Aldrich, St Louis, MO, United States) or anti-mouse CD3ε (plate coated at 5 μg/ml, Bio X cell, Lebanon, NH, United States) plus 2 μg/ml soluble anti-mouse CD28 (Bio X cell) for 24 h h, or 50 ng/ml phorbol 12-myristate 13-acetate (PMA) (Sigma-Aldrich) and 1 μg/ml ionomycin (Sigma-Aldrich) for 6 h. For the induction of IL-17, the splenocytes were stimulated with either non-pathogenic T helper 17 (Th17) stimuli (20 ng/ml IL-6 (BioLegend) plus 3 ng/ml TGFβ 1 (R&D System Inc., Minneapolis, MN, United States) and 1 μg/ml anti-CD3) or pathogenic Th17 stimuli (20 ng/ml IL-6 plus 10 ng/ml IL-1β (BioLegend), 10 ng/ml IL-23 (Invitrogen) and 1 μg/ml anti-CD3) for 72 h. The cell culture supernatants were collected for ELISA assay.

### *In Vitro* Transfection of Antagomir

As previously described (Krützfeldt et al., 2005; Dai et al., 2016), the specific antagomirs against certain miR-182, miR-183, and miR-96 were designed based on mature murine miRNA sequences from miRbase (http://www.mirbase.org/) and shown in Table 1. The scramble control antagomir (Stittrich et al., 2010) and specific antagomirs were synthesized by GE Dharmacon (Lafayette, CO, United States). Antagomirs were delivered into splenic lymphocytes using a serum-free Accell siRNA delivery medium (GE Dharmacon) as we reported previously (Dai et al., 2016). Twenty-four hours after antagomir treatment, the cells were stimulated with concanavalin A (Con A; 5 μg/ml) or LPS (500 ng/ml) for 24 h or 48 h, the culture supernatants were collected to measure the cytokine levels by ELISA. The cell pellets were collected for Western blot.

**TABLE 1:** Scrambled control and specific miR-183, -96, -182 antagomirs sequences.

Antagomir ID	Sequences
Scrambled Control	5′mU (*)mC (*)mAmCmGmCmAmGmAmUmUmCmAmUmAmA (*)mC (*)mG (*)mU (*)-3′-Chl
Antagomir-183	5′ mA (*)mG (*)mUmG mAmAmU mUmCmU mAmCmC mAmGmUmGmCmC (*)mA (*) mU (*)mA (*) -3′-Chl
Antagomir-96	5′ mA (*)mG (*)mCmA mAmAmA mAmUmG mUmGmC mUmAmG mUmGmC mC (*)mA (*)mA (*) mA (*) -3′-Chl
Antagomir-182	5′mC (*)mG (*)mGmUmGmUmGmAmGmUmUmCmUmAmCmCmAmUmUmGmCmC(*) mA (*)mA (*)mA (*)-3′-Chl

All ribonucleotides are 2′-O-methyl modified (mN). (*) represents a phosphorothioate modification of the backbone. A cholesterol molecule was added at the 3′ end (-3′-Chl).

### RNA Extraction

As we reported previously (Dai et al., 2016; Wang Z. et al., 2018; Dai et al., 2019), total RNA containing small RNA was extracted from cells using the miRNeasy Mini Kit (Qiagen) following the manufacturer’s protocol. On-column DNA digestion was performed to remove any residual DNA in the RNA samples during RNA extraction. Concentration and purity of RNA were determined by NANODROP 2000 spectrophotometer (Thermo Fisher Scientific, United States) and samples with the OD260/280 ratio at around 2.0 were used for TaqMan miRNA Assay and RT-qPCR analysis.

### Quantitative RT-PCR Analysis of miRNA and mRNA

As we previously reported (Dai et al., 2010; Dai et al., 2016; Wang Z. et al., 2018; Dai et al., 2019), TaqMan microRNA assays (Applied Biosystems, Waltham, MA, United States) were used to quantify miR-183C miRNAs (miR-182 ID 002599; miR-183 ID 00269; miR-96 ID 000186; snoRNA202 ID 001232, Applied Biosystems) expression levels according to the manufacturer’s instruction. For miRNA analysis, RNA samples were reverse transcribed with TaqMan microRNA reverse transcription kit, followed by quantitative real-time PCR (qPCR) with TaqMan miRNA assay reagent. For RT-qPCR of protein-coding genes, total RNA samples were reverse transcribed using High-Capacity cDNA Reverse Transcription Kit (Applied Biosystems, Waltham, MA, United States) followed by qPCR using Power SYBR green PCR master mix (Applied Biosystems). The qPCR reactions were performed with a 7,500 Fast Real-Time PCR system (Applied Biosystems, Waltham, MA, United States). The relative expression level of miRNAs and mRNAs was normalized to endogenous small nucleolar RNA 202 (snoRNA202) and house-keeping gene β–actin respectively and calculated relative expression level using the 2^−ΔΔCt^ method. The primers for quantitative RT-PCR were as follows, Foxo1: (Forward) 5′-CGT​GCC​CTA​CTT​CAA​GGA​TAA​G-3′, (Reverse) 5′-GCA​CTC​GAA​TAA​ACT​TGC​TGT​G-3'; β–actin: (Forward) 5′-CGC​GAG​CAC​AGC​TTC​TT-3′, (Reverse) 5′-GCA​GCG​ATA​TCG​TCA​TCC​AT-3'.

### ELISA

The production level of interferon-gamma (IFNγ), interleukin 6 (IL-6), and interleukin 17 (IL-17) in cell culture supernatant were detected ELISA kits from BioLegend and ThermoFisher Scientific) respectively. Serum levels of IL-6, IFNγ, IL-17, TNF-a, and IL-10 were determined by the SP-X (previously Ciraplex^®^) multiplex Chemiluminescent Assay kit (Quanterix, Billerica, MA, United States) as we previously reported (Dai et al., 2019; Edwards et al., 2020). The image of the chemiluminescent array plate was captured with the Aushon Cirascan™ Imaging system (Aushon BioSystems, InC., Billerica, MA, United States) and the image data was processed with Cirasoft software.

Anti-dsDNA autoantibody ELISA was performed to detect serum anti-dsDNA autoantibody production levels as we previously reported (Dai et al., 2010; Edwards et al., 2017). The plates were read at 380 nm with a Versa microplate reader (Molecular Devices).

### Flow Cytometry

The relative proportion of subsets of immune cells was quantified by flow cytometric analysis with specific cell surface markers as we previously reported (Dai et al., 2019; Dai et al., 2020). Briefly, the freshly-isolated splenocytes were blocked with purified anti-mouse CD16/CD32 antibody (eBiosciences/ThermoFisher Scientific), and stained with cell surface markers in MACS buffer (Phosphate-buffered saline (PBS, pH 7.2) supplemented with 0.5% bovine serum albumin (BSA) and 2 mM EDTA) for 15–20 min at 4°C. After washing, the stained cells were resuspended in 100 μl MACS buffer for flow cytometry analysis. Anti-CD3-FITC, anti-CD4-eFluor 450, anti-CD8a-Percp-Cy5.5, anti-CD19-Percp-Cy5.5, anti-B220-PE, anti-CD25-Percp/Cy5.5, anti-Foxp3-APC, and anti-GL7-eFluor660 were purchased from eBiosciences/ThermoFisher Scientific. Anti-CXCR5-APC/Cy7, anti-CD138-APC (PE), anti-IgD-PE were obtained from BioLegend, anti-PD1-BV421 was obtained from BD biosciences. FACSAria Flow cytometer (BD Biosciences, San Jose, CA) was used for stained cell profiling. Data were analyzed by FlowJo software (FlowJo LLC, BD Life Sciences).

### Western Blot Analysis

Western blotting was performed by following the lab-established protocol (Dai et al., 2007; Dai et al., 2009). Briefly, whole cell extracts were prepared by lysing the cell pellets with CelLytic™ M Cell Lysis Reagent (Sigma-Aldrich). Protein lysates were separated by 4%–15% Criterion™ TGX™ Precast sodium dodecyl sulfate-polyacrylamide gel electrophoresis (SDS-PAGE) (Bio-Rad Laboratories, Inc.) and then transferred onto a polyvinylidene difluoride (PVDF) membrane. Membranes were blocked with 5% non-fat milk in PBST, incubated with a primary antibody, and subsequently a secondary horseradish peroxidase-conjugated antibody. After applying ECL plus western blot substrate (GE Healthcare, Cleveland, OH, United States), the blot images were captured with iBright CL1000 Imaging System (ThermoFisher Scientific). The Foxo1 antibody was purchased from Cell Signaling Technology, Inc. United States. β–actin antibody (Santa Cruz Biotechnology) was used as a reference protein.

### Renal Histopathology and Renal Immunofluorescence

Renal histopathology and immunofluorescence analysis were performed as we previously reported (Dai et al., 2013; Edwards et al., 2017; Edwards et al., 2020). Frozen section slides and hematoxylin and eosin (H&E) stained slides were prepared by the histopathology laboratory at VMCVM, Virginia Tech. The H&E renal sections were assessed by a board-certified pathologist in a blinded fashion. A grade of 0–4 (0 = perfect, no change; 1 = minimal; 2 = moderate, 3 = marked; 4 = severe) was given to reflect the glomerular, tubular, interstitium, and vessel inflammation and lesions, respectively.

The OCT frozen kidneys were cut to 5 μM sections in the histopathology laboratory at VMCVM. The frozen slide sections were stored at −80°C for immunofluorescent staining. Briefly, the frozen slides were air-dried, fixed with cold acetone for 10 min at room temperature, followed by blocking with PBS containing 1% BSA (Fisher) and anti-mouse CD16/32 for 20–30 min. The slides were then incubated with FITC-conjugated IgG (eBioscience, San Diego, CA, United States) and PE-conjugated Complement Component C3 (Cedarlane, Burlington, NC, United States) for 1–2 h at room temperature in a dark chamber. The stained slides were mounted with coverslips using Prolong Gold anti-fade reagent with DAPI (Invitrogen, Grand Island, NY, United States). The stained kidney sections were assessed for IgG and C3 deposition under a fluorescent microscope. Fiji/ImageJ image processing program (Schindelin et al., 2015) was used to measure the fluorescent intensity of the selected area as we previously described (Edwards et al., 2017; Edwards et al., 2020).

### siRNA Transfection

The Foxo1 specific Dicer substrate siRNA (Foxo1 siRNA) and negative control DsiRNA (NC) were purchased from Integrated DNA Technologies (Coralville, IA, United States) and reconstituted with distilled water to a stock concentration at 10 μM. The DsiRNAs were transfected into splenic lymphocytes with Lipofectamine RNAiMAX transfection reagent (ThermoFisher Scientific) per the manufacturer’s protocol as we recently reported (Dai et al., 2020). Forty-eight hours after transfection, the transfected cells were stimulated with anti-mouse CD3ε (plate coated at 5 μg/ml) plus 2 μg/ml soluble anti-mouse CD28 (Bio X cell) for 24 h h, or 50 ng/ml phorbol 12-myristate 13-acetate (PMA) plus 1 μg/ml ionomycin for 6 h. The cell culture supernatants were collected for ELISA assay.

### Statistical Analysis

All the values in the graphs were given as means ± SD. Unpaired student t-test and one-way ANOVA with Tukey-Kramer all pair’s comparisons were performed to determine the statistical significance for two or multiple group comparisons, respectively. “*,” “**,” and “***” indicate *p* < 0.05, *p* < 0.01, and *p* < 0.001, respectively. The graphic presentations of data were performed in GraphPad Prism software (v.8.10 for Windows).

## Results

### Inhibition of Either miR-182 Alone or miR-183C *In Vitro* in Splenocytes Reduces Inflammatory Cytokines IFNγ and IL-6 Expression Level

**FIGURE 1:**
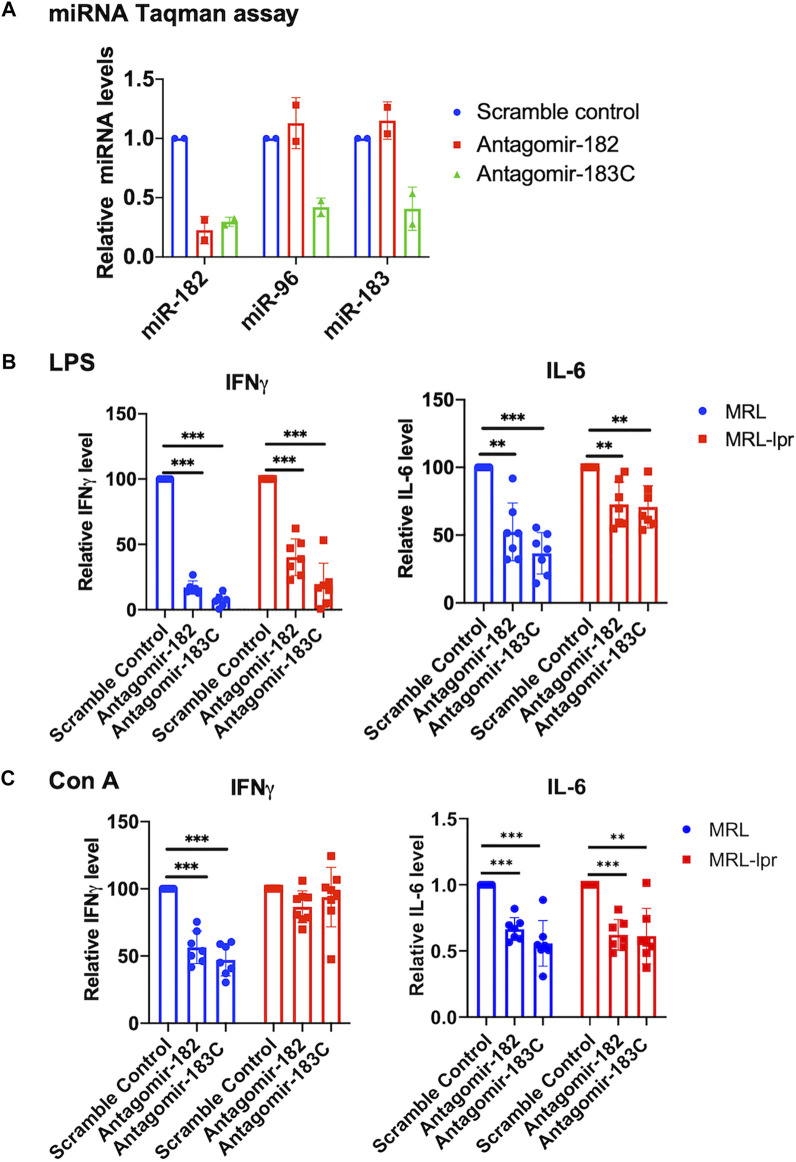
*In vitro* inhibition of miR-182 alone and miR-183C miRNAs in splenocytes reduces inflammatory cytokines IFNγ and IL-6 expression level. **(A)** Antagomir-182, -96, -183 inhibited the respective miRNA efficiently and specifically. Freshly-isolated splenocytes from MRL/lpr mice were transfected with either scramble control, antagomir-182, or the mixture of antagomir-182, -96, and -183 (antagomir-183C). Twenty-four hours after transfection, the cells were collected for TaqMan miRNA assays. The graph showed means ± SD (*n* = 2 each). **(B,C)** Inhibition of miR-182 or miR-183C reduced IFNγ and IL-6 production in LPS or Con A-activated splenocytes. Freshly-isolated splenocytes from MRL and autoimmune-prone MRL/lpr mice at 14–15 weeks of age were treated with control antagomir and specific antagomir for 24 h, and then stimulated with LPS (500 ng/ml) for 48 h or Con A (5 µg/ml) for 24 h. The IFNγ and IL-6 levels in the culture supernatant were measured by ELISA. The graphs showed means ± SD (*n* ≥ 6). The cytokine level in specific antagomir treated samples were shown as the percentage of paired scrambled control antagomir treated cells. Paired student *t* tests were performed (scrambled control vs*.* antagomir-182 or antagomir-183C); **, *p* < 0.01; and ***, *p* < 0.001.

We have previously reported a significant upregulation of miR-183C miRNAs in the splenocytes of different murine lupus models (Dai et al., 2010). To determine whether miR-183C miRNAs play a role in regulating lupus-related inflammatory cytokines such as IFNγ and IL-6, we inhibited miR-182 alone or miR-183C miRNAs with specific antagomirs *in vitro* in splenocytes (Figure 1A). The antagomir-treated cells from MRL (control) and MRL/lpr (lupus-prone) were stimulated with two different stimuli, LPS and Con A, which activated immune cells to elicit cytokine production via distinct signaling pathways. As indicated, the inhibition of miR-182 alone or the miR-183C whole cluster significantly suppressed LPS-induced IFNγ and IL-6 production in both MRL and MRL/lpr splenocytes (Figure 1B). In Con A-stimulated cells, miR-182 and miR-183C inhibition significantly reduced both IFNγ and IL-6 in MRL splenocytes, while in MRL/lpr splenocytes only IL-6 was significantly reduced (Figure 1C). The antagomir treatment did not affect the cell viability (Supplementary Figure S2A). We also noticed that silencing miR-182, but not miR-96 and miR-183 alone, had a similar suppression on IFNγ and IL-6 production as silencing the three miR-183C miRNAs (Supplementary Figure S2B). This suggests that of the three miR-183C miRNAs, miR-182 likely plays a major role in regulating inflammatory cytokine production.

### Conditional Deletion of miR-183C Significantly Suppresses Anti-dsDNA Autoantibodies in B6/lpr Mice

**FIGURE 2:**
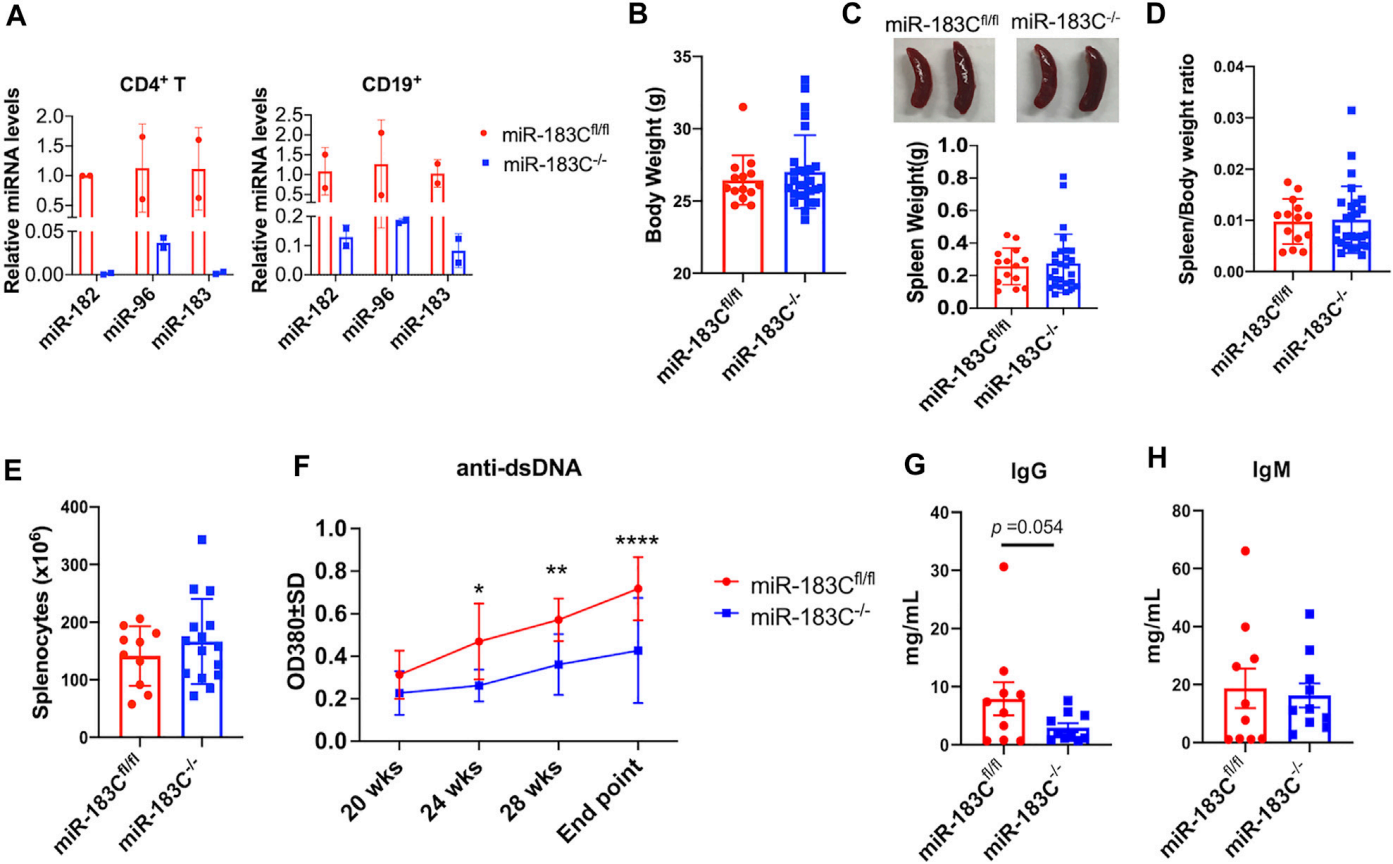
Analysis of the effect of miR-183C deletion on body weight, spleen weight, and antibody production. **(A)** miRNA TaqMan assay was performed to validate the deletion of miR-182, -96, -183 miRNAs in purified CD4^+^ T and CD19^+^ B cells from conditional miR-183C^−/−^B6/lpr (miR-183C^−/−^) mice. Data are presented as means ± SD (*n* = 2 each). **(B–E)** Deletion of miR-183C had no obvious effect on body weight **(B)**, spleen weight **(C)**, spleen/body weight ratio **(D)**, and splenocyte counts **(E)**. The miR-183C^−/−^ and control miR-183C^fl/fl^B6/lpr (miR-183C^fl/fl^) mice were euthanized at 29–32 weeks of age (end point) for the assay. Data are displayed as means ± SD (*n* ≥ 10). **(F)** Deletion of miR-183C miRNAs significantly reduced serum anti-dsDNA autoantibodies. The serum anti-dsDNA autoantibody levels in miR-183C^−/−^ and control miR-183C^fl/fl^ mice were monitored every 4 weeks starting at 20 weeks of age. The graph showed means ± SD (*n* = 6 for miR-183C^−/−^ mice at different time points except *n* = 12 for the end point; *n* = 10 for 183C^fl/fl^ mice at different time points except *n* = 15 for the end point). **(G,H)** Evaluation of serum levels of total IgG **(G)** and IgM **(H)** antibodies in miR-183C^−/−^ and control mice at the end point. Graphs showed means ± SD (*n* = 10 each). Unpaired student *t* tests were performed (miR-183C^fl/fl^ vs*.* miR-183C^−/−^); *, *p* < 0.05; **, *p* < 0.01.

To further investigate the role of upregulated miR-183C cluster miRNAs *in vivo* in autoinflammation-prone mice, we generated miR-183C^−/−^B6/lpr mice with conditional deletion of miR-183C miRNA cluster in both T and B lymphocytes (Figure 2A). As previously reported (de Boer et al., 2003; Siegemund et al., 2015), the hCD2-iCre deleted the floxed-target gene in both T and B lymphocytes. Nevertheless, the deletion efficiency in CD4^+^ T (about 99% reduction) is higher than that in CD19^+^ B (about 90% reduction) cells (Figure 2A). After the mice reached 20 weeks of age, blood samples were collected at 4 weeks intervals to monitor the serum level of anti-double strand DNA (anti-dsDNA) autoantibody in miR-183C^−/−^B6/lpr and control miR183C^fl/fl^B6/lpr mice. The mice were euthanized at 29–32 weeks of age (endpoint) for the experimental analysis. Conditional deletion of miR-183C had no obvious effect on the body weight, spleen weight, and absolute splenocyte counts (Figures 2B–E). Impressively, we found that miR-183C deletion significantly reduced serum anti-dsDNA autoantibody level in miR-183C^−/−^B6/lpr mice compared to age-matched miR183C^fl/fl^B6/lpr control mice (Figure 2F). There was a trend of reduced total IgG in miR-183C^−/−^B6/lpr mice (*p* = 0.054, Figure 2G). There was no difference in the serum IgM levels (Figure 2H).

**FIGURE 3:**
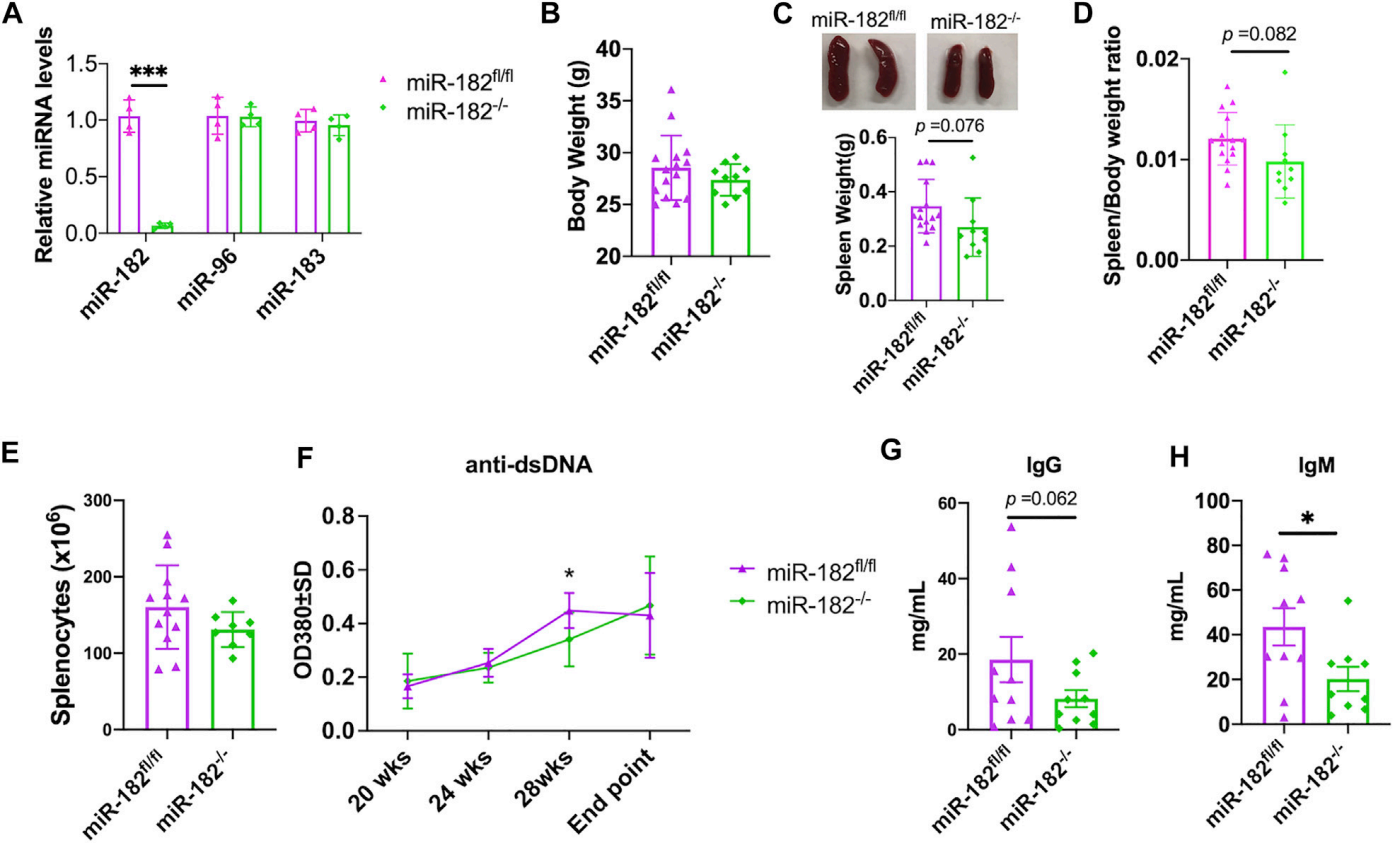
Analysis of the effect of miR-182 deletion on body weight, spleen weight, and antibody production. **(A)** miRNA TaqMan assay was performed to validate the specific deletion of miR-182, but not miR-96 and miR-183 in splenocytes of miR-182^−/−^B6/lpr (miR-182^−/−^) mice. The graph showed means ± SD (*n* = 4 each). **(B–E)** Deletion of miR-182 alone had no obvious effect on body weight **(B)** and splenocyte counts **(E)**, but led a trend of reduction of spleen weight **(C)** and spleen/body weight ratio **(D)**. The miR-182^−/−^ and control miR-182^fl/fl^B6/lpr (miR-182^fl/fl^) mice were euthanized at 29–32 weeks of age (end point) for sample collections. Graphs showed means ± SD (*n* ≥ 8). **(F)** Deletion of miR-182 had minimal effect on serum anti-dsDNA autoantibodies. The serum anti-dsDNA autoantibody levels in miR-182^−/−^ and miR-182^fl/fl^ mice were monitored every 4 weeks starting at 20 weeks of age. Data were shown as means ± SD (*n* = 6 for miR-182^−/−^ mice at different time points except *n* = 15 for the end point; *n* = 7 for miR-182^fl/fl^ mice at different time points except *n* = 15 for the end point) **(G,H)** Evaluation of serum levels of total IgG **(G)** and IgM **(H)** antibodies in miR-182^−/−^ and miR-182^fl/fl^ mice at the end point. Graphs showed means ± SD (*n* = 10 each). Unpaired student *t* tests were performed (miR-182^fl/fl^ vs*.* miR-182^−/−^); *, *p* < 0.05; ***, *p* < 0.001.

The *in vitro* antagomir inhibition data indicated that inhibition miR-182 alone and inhibition of the whole miR-183C had a similar suppressive effect on activation induced inflammatory cytokines production (Figure 1). We, therefore, derived miR-182^−/−^B6/lpr to determine whether deletion of miR-182 alone *in vivo* has a similar effect as the deletion of whole cluster *in vivo* in B6/lpr mice. As expected, miR-182 was specifically deleted in the splenocytes of miR-182^−/−^B6/lpr mice (Figure 3A). There was no change in the expression of miR-96 and miR-183 in the splenocytes of miR-182^−/−^B6/lpr mice when compared to control miR182^fl/fl^B6/lpr mice (Figure 3A). Conditional deletion of miR-182 alone did not affect the body weight (Figure 3B). There was a trend of decreased spleen weight (*p* = 0.076) and spleen/body weight ratio (*p* = 0.082) in miR-182^−/−^B6/lpr mice when compared to controls (Figures 3C,D). However, there was no obvious change in the absolute splenocytes count in miR-182^−/−^B6/lpr compared to control miR-182^fl/fl^B6/lpr mice (Figure 3E). We only observed a reduction of serum anti-dsDNA levels in miR-182^−/−^B6/lpr mice at 28 weeks of age compared to age-matched controls (Figure 3F). Deletion of miR-182 significantly decreased serum IgM levels and demonstrated a trend of reduced total IgG (*p* = 0.062) in B6/lpr mice (Figures 3G,H).

### Conditional Deletion of miR-183C or miR-182 Reduces IgG Immune Complex Deposition in the Kidneys of B6/lpr Mice

**FIGURE 4:**
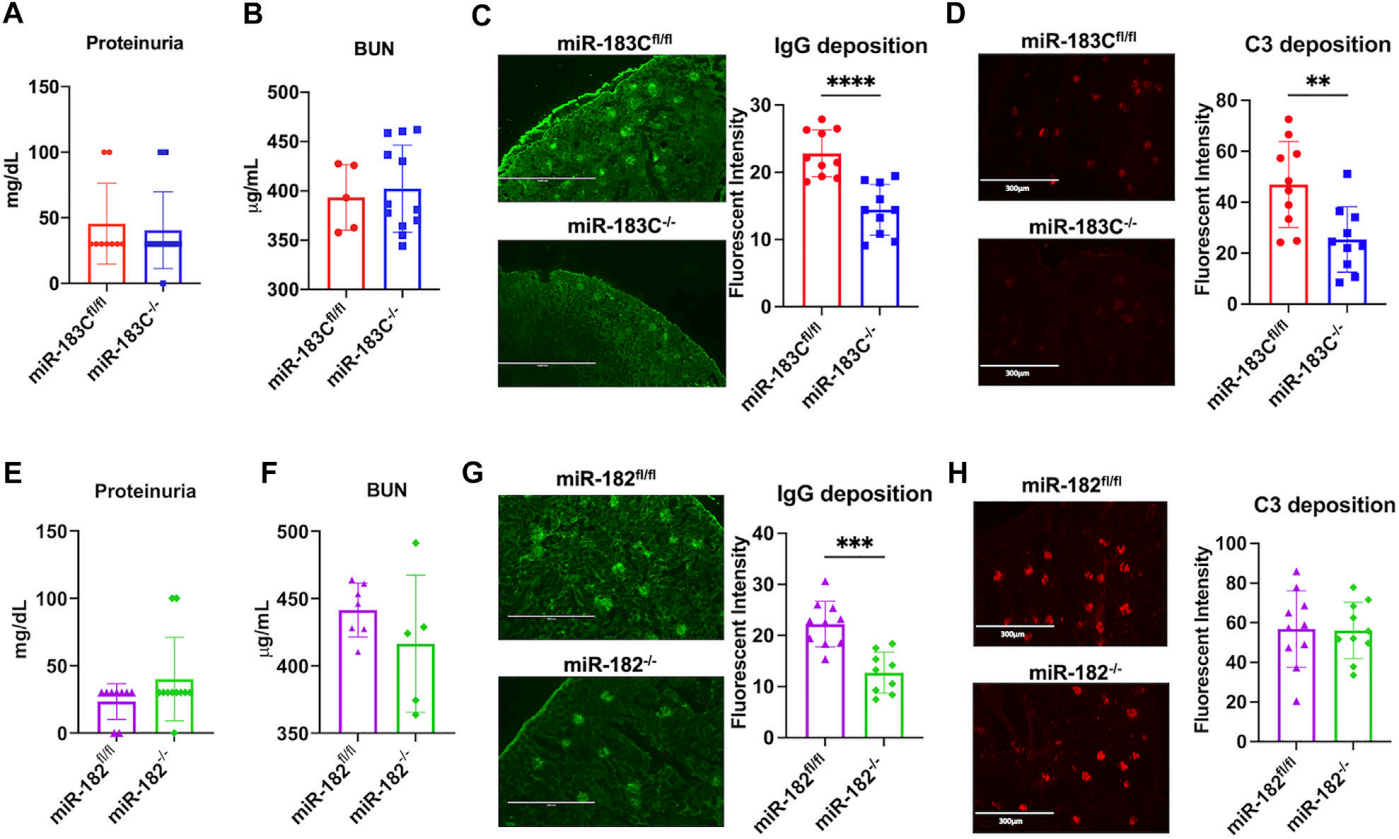
Deletion of either miR-183C or miR-182 alone reduces IgG antibody deposition in kidney. **(A,B)** Summary graphs show no significant difference in the proteinuria **(A)** and blood urea nitrogen (BUN, **(B)** levels between miR-183C^−/−^B6/lpr and control miR-183C^fl/fl^B6/lpr mice. Graphs showed means ± SD (*n* ≥ 5) **(C,D)** Representing IF images and summary data show significantly reduced IgG **(C)** and complement C3 **(D)** immune complex deposition in the kidneys of miR-183C^−/−^. Graphs showed means ± SD (*n* = 10 each) **(E,F)** Summary graphs show no significant difference in the proteinuria **(E)** and blood urea nitrogen (BUN, **(F)**) levels between miR-182^−/−^B6/lpr and control miR-182^fl/fl^B6/lpr mice. Graphs showed means ± SD (*n* ≥ 5) **(G,H)** Representing images and summary data show significantly reduced IgG **(G)** and complement C3 **(H)** immune complex deposition in the kidneys of miR-182^−/−^. Graphs showed means ± SD (*n* = 10 each). Unpaired student t tests were performed (miR-183C^fl/fl^ vs*.* miR-183C^−/-^, miR-182^fl/fl^ vs*.* miR-182^−/−^); *, *p* < 0.05; and **, *p* < 0.01.

The effect of miR-183C deletion on the renal function of B6/lpr was determined by examining proteinuria and blood urea nitrogen (BUN) levels. There was no difference in the proteinuria or BUN levels between miR-183C^−/−^B6/lpr and control miR-183C^fl/fl^B6/lpr mice (Figures 4A,B). The renal histopathology analysis also showed no obvious difference in the kidney inflammation and damages in the miR-183C^−/−^B6/lpr mice compared to control miR-183C^fl/fl^B6/lpr mice (Table 2). However, we observed a significant reduction of IgG and complement component C3 immunocomplex deposition in the kidneys of miR-183C^−/−^B6/lpr mice compared to age-matched control mice (Figures 4C,D). We also examined the effect of miR-182 deletion on renal pathology and function in B6/lpr mice. Similar to the data with miR-183C deletion, deletion of miR-182 had no effect on renal histopathology, proteinuria levels, serum BUN levels and C3 immunocomplex deposition in the kidneys in B6/lpr mice (Figures 4E,F,H), but significantly reduced IgG immune complex deposition in the kidneys of B6/lpr mice (Figure 4G).

**TABLE 2:** Renal histopathological scores.

Strains	Glomeruli	Tubules	Interstitium	Vessels	Kidney Scores
miR183C^fl/fl^B6/lpr (*n* = 8)	1.13 ± 0.64	0.13 ± 0.35	0 ± 0	0.63 ± 0.52	1.88 ± 1.13
miR183C^−/−^B6/lpr (*n* = 11)	0.73 ± 0.65	0 ± 0	0 ± 0	0.91 ± 0.83	1.64 ± 1.29
miR182^fl/fl^B6/lpr (*n* = 9)	1 ± 0.5	0 ± 0	0.22 ± 0.44	0.78 ± 0.67	2 ± 1.41
miR182^−/−^B6/lpr (*n* = 8)	1.25 ± 1.04	0 ± 0	0 ± 0	0.38 ± 0.52	1.63 ± 1.19

Evaluation and scoring of the formalin-fixed renal tissue section. H&E-stained slides were evaluated by veterinary pathologists in a blinded fashion. Scores were averaged for each group and each structure. Data are shown as Mean ± SD.

### Conditional Deletion of miR-183C or miR-182 Does Not Change Lymphocyte Composition in the Spleens of B6/lpr Mice

**FIGURE 5:**
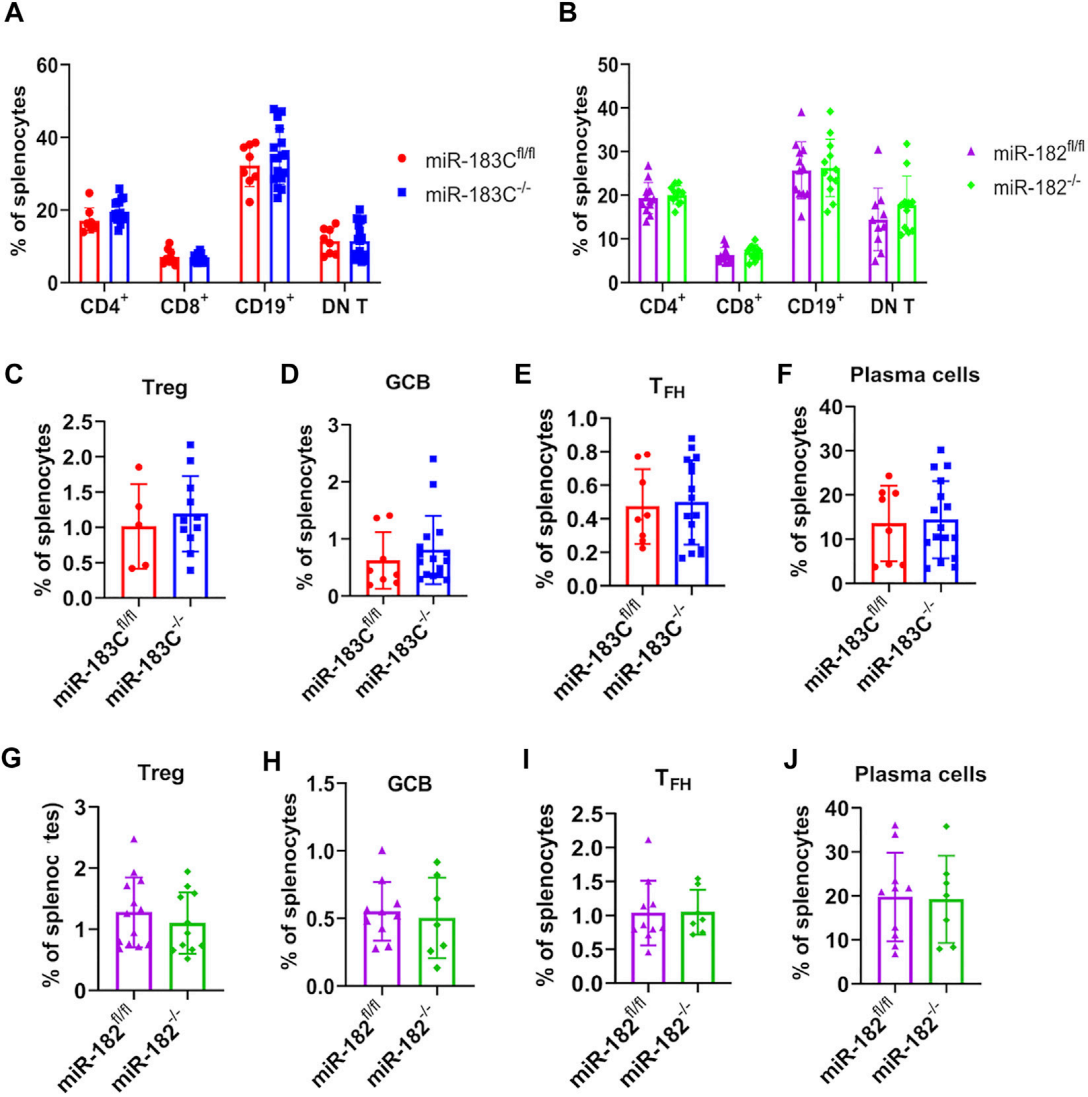
Evaluate the effect of miR-182 and miR-183C deletion on splenic lymphocyte composition in B6/lpr mice. The different lymphocyte subsets in the freshly-isolated splenocytes were analyzed by flow cytometry. Please see Supplementary Figure S3 for the gating of different immune cell subsets in the splenocytes. **(A,B)** Summary data of the frequency of CD4^+^ T cells, CD8^+^ T cells, CD19^+^ cells, and CD3^+^B220^+^CD4^−^CD8^−^ double negative T cells (DN T cells) in the splenocytes of miR-183C^−/−^ and control miR-183C^fl/fl^ B6/lpr **(A)**, miR-182^−/−^ and control miR-182^fl/fl^ B6/lpr **(B)**. The graphs show means ± SD (*n* ≥ 8). **(C–J)** Summary data of the frequency of differentiated CD4^+^CD25^+^Foxp3^+^ **(C,G)**, CD19^+^GL7^+^IgD^−^ GCB **(D,H)**, CD3^+^CD4^+^CXCR5^+^PD1^+^ T_FH_ **(E,I)**, CD19^−^CD138^+^ plasma **(F,J)** cells in the splenocytes of miR-183C^−/−^ and miR-183C^fl/fl^ **(C–F)**, miR-182^−/−^ and miR-182^fl/fl^ **(G–J).** The graphs show means ± SD (*n* ≥ 5). Unpaired student *t* tests were performed (miR-183C^fl/fl^ vs*.* miR-183C^−/−^, miR-182^fl/fl^ vs*.* miR-182^−/−^) for statistical analysis. No significant differences were observed in the difference immune cell subsets between knockout and control mice.

Previous studies have reported that deletion of miR-182 *in vivo* had no obvious effects on adaptive immune cell development in normal B6 mice (Pucella et al., 2015; Ichiyama and Dong, 2019). Flow cytometric evaluation was performed to determine whether miR-182 and miR-183C deletion affected immune cell composition in B6/lpr mice (Supplementary Figure S3). Consistently, we found that in the autoinflammatory-prone B6/lpr mice, conditional deletion of either miR-182 alone or miR-183C cluster in lymphocytes also did not change the adaptive immune cell development and lymphocyte subset composition in the spleens (Figures 5A,B). The development of CD4^−^CD8^−^CD3^+^B220^+^ double-negative T cells (DN T) is a specific characteristic cell type in *lpr* lupus mice (Morse et al., 1985; Giese et al., 1993; Hamad et al., 2003). There were no obvious differences in the percentage of DN T cells in the splenocytes between the knockout and control B6/*lpr* mice (Figures 5A,B). While the previous study has shown that miR-182 regulates Treg cell development during murine EAE development (Wan et al., 2016), we did not observe any notable change of Tregs percentage in the splenocytes in either the miR-183C^−/−^B6/lpr or the miR-182^−/−^B6/lpr mice compared to their respective controls (Figures 5C,G). Furthermore, we found that conditional deletion of miR-183C or miR-182 did not affect the differentiation of germinal center B cells (GCB), follicular helper T cells (T_FH_), or plasma cells in the spleens. The percentages of GCB, T_FH_, and plasma cells in the splenocytes of the miR-183C^−/−^B6/lpr (Figures 5D–F) and the miR-182^−/−^B6/lpr (Figures 5H–J) mice were similar to their respective controls.

### Deletion of miR-183C and miR-182 *In Vivo* in B6/lpr Mice Significantly Reduces IFNγ, but Not IL-6 Production in *In Vitro* Activated Splenocytes

**FIGURE 6:**
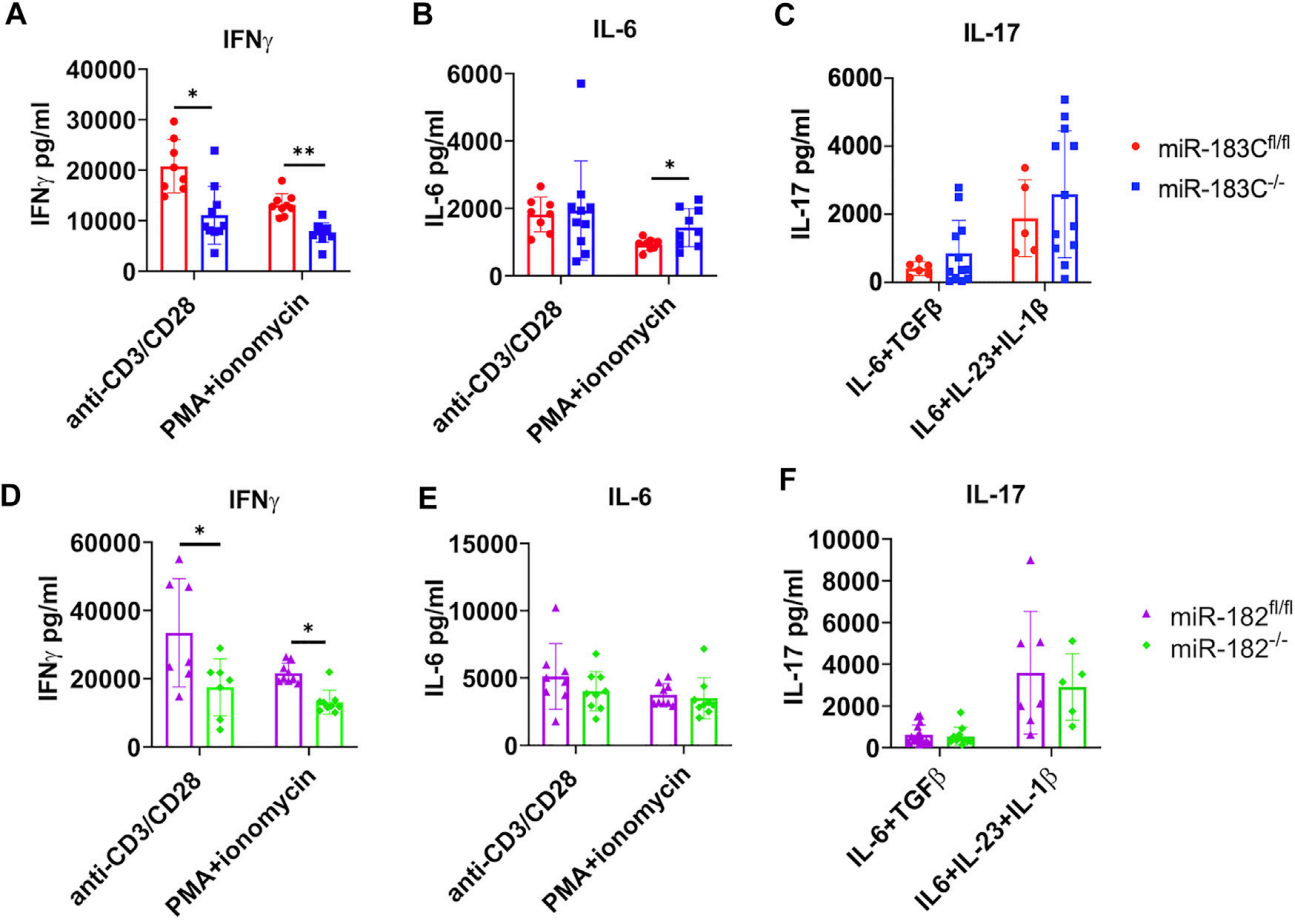
Exam the effect of miR-182 and miR-183C deletion on IFNγ, IL-6, and IL-17 production in *in vitro* activated splenocytes with different stimuli. The freshly isolated splenocytes from miR-183C^−/-^B6/lpr **(A–C)**, miR-182^−/−^B6/lpr **(D–F)** and their respective littermate control miR-miR-183C^fl/fl^ and miR-182^fl/fl^B6/lpr mice were stimulated with anti-CD3 plus anti-CD28 for 24 h, PMA plus ionomycin for 6 h, non-pathogenic Th17 stimuli or pathogenic Th17 stimuli for 72 h. The production specific cytokines in culture supernatant were measured by ELISA. **(A–C)** Summary graphs show deletion of miR183C *in vivo* in B6/lpr mice suppressed IFNγ, but not IL-6 and IL-17 in splenocytes following *in vitro* stimulation with specific stimuli. **(D–F)** Summary graphs show deletion of miR182 alone *in vivo* in B6/lpr mice suppressed IFNγ, but not IL-6 and IL-17 in *in vitro* activated splenocytes with specific stimuli. The graphs were shown as means ± SD (*n* ≥ 5). Unpaired student t tests were performed (miR-183C^fl/fl^ vs*.* miR-183C^−/−^, miR-182^fl/fl^ vs*.* miR-182^−/−^) for statistical analysis; *, *p* < 0.05; **, *p* < 0.01; and ***, *p* < 0.005.

We showed that *in vitro* inhibition of either miR-182 alone or miR-183C significantly suppressed IFNγ and IL-6 production in activated splenocytes (Figure 1). We then checked IFNγ and IL-6 cytokine production in *in vitro* activated splenocytes from miR-183C^−/−^B6/lpr and control miR-183C^fl/fl^B6/*lpr* mice. We found that in response to either anti-CD3 plus anti-CD28 or PMA plus ionomycin stimulation, there was a significantly lower IFNγ production in splenocytes from miR-183C^−/−^B6/lpr and miR-182^−/−^B6/lpr mice compared to splenocytes from their respective control mice (Figures 6A,B). *In vivo* deletion of miR-183C or miR-182 alone had no effect on IL-6 production in splenocytes in response to anti-CD3 plus anti-CD28 stimulation (Figures 6B,E). There was an increase of IL-6 in PMA plus ionomycin-activated splenocytes from miR-183C^−/−^B6/lpr mice, but not from miR-182^−/−^B6/lpr compared to their respective controls (Figures 6B,E). A recent study revealed that miR-183C was highly induced by IL-6-STAT3 signaling to promote the differentiation and function of pathogenic Th17 cells (Ichiyama et al., 2016). We found that deletion of miR-183C or miR-182 *in vivo* did not affect IL-17 production in B6/lpr splenocytes in response to either non-pathogenic (IL-6 plus TGFβ1) or pathogenic (IL-6, IL-23 plus IL-1β) Th17 stimuli (Figures 6C,F). *In vitro* inhibition of either miR-183C or miR-182 alone suppressed LPS-induced IFNγ and IL-6 production in splenocytes from MRL and MRL/lpr mice (Figure 1). However, we found that deletion of miR-183C *in vivo* did not affect LPS-induced IFNγ and IL-6 production, and the deletion of miR-182 only inhibited LPS-induced IFNγ, but not IL-6 in splenocytes from B6/lpr mice (data not shown).

### miR-183C miRNAs Regulate Inflammatory Cytokine IFNγ *via* Targeting Foxo1

**FIGURE 7:**
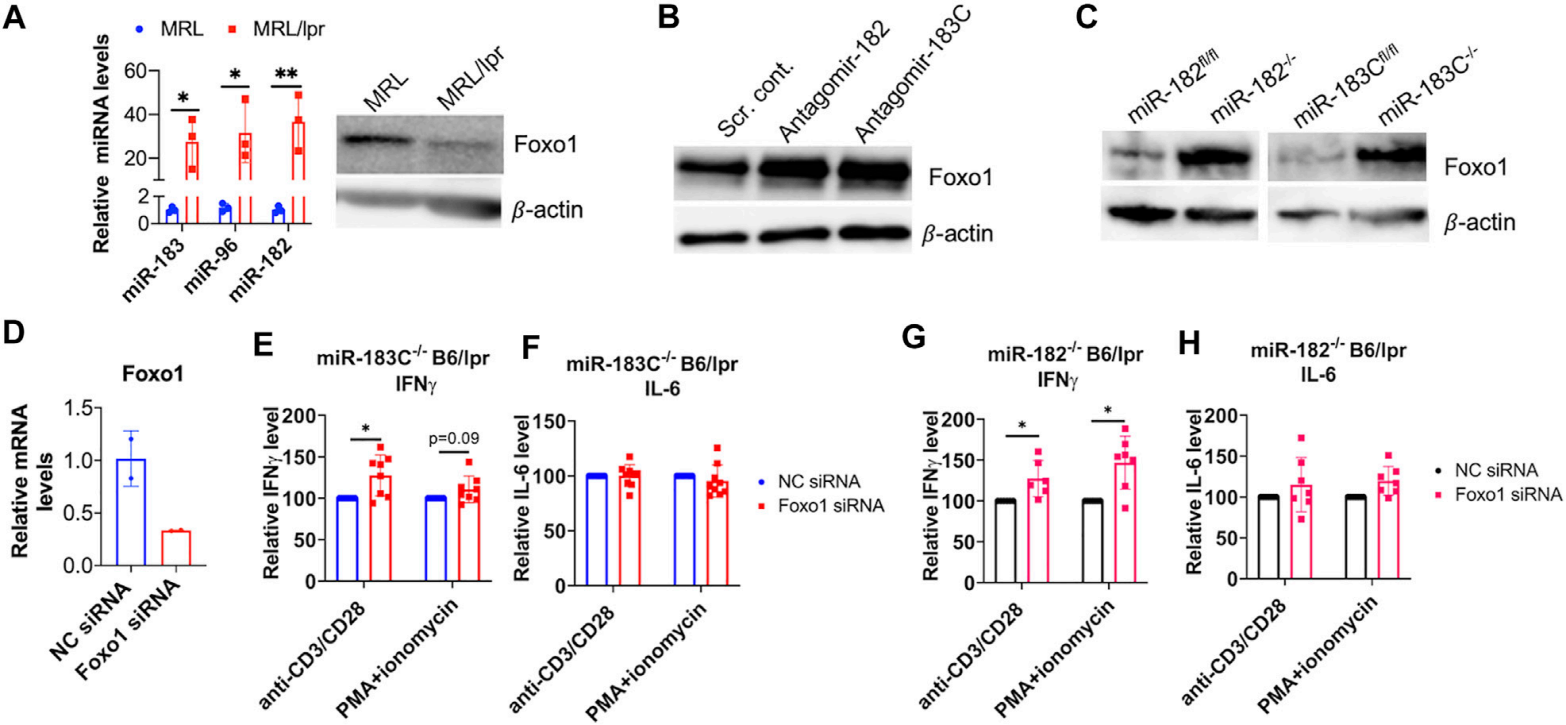
miR-183C and miR-182 miRNAs regulate IFNγ production via targeting Foxo1. **(A)** An inverse relationship between miR-183 miRNAs expression of Foxo1 protein expression in control MRL and autoimmune-prone MRL/lpr mice splenocytes. The left graph showed relative miR-183, -96, -182 miRNAs expression in the splenocytes of MRL and MRL/lpr mice (14–15 weeks of age). The graph showed means ± SD (*n* = 3 each). Unpaired student t -test (MRL vs. MRL/lpr); *, *p* < 0.05; **, *p* < 0.01. The right panel showed western blotting of Foxo1 and β-actin (loading control) protein expression in MRL and MRL/lpr splenocytes. The representative western blotting image from three independent experiment was shown. **(B)** Increased Foxo1 protein in antagomir-182 or antagomir-183C treated splenocytes from MRL/lpr mice. The representative western blotting image from two independent experiments was shown. **(C)** Increased Foxo1 protein expression in splenic CD4^+^ T cells from miR-183C^−/−^B6/lpr (miR-183C^−/−^), miR-182^−/−^B6/lpr (miR-182^−/−^) mice when compared to the cells from their respective control miR-183C^fl/fl^B6/lpr (miR-183C^fl/fl^) and miR-182^fl/fl^B6/lpr (miR-182^fl/fl^) mice. The representative western blotting image from at least three independent experiments was shown. **(D)** Reduced Foxo1 mRNA expression in Foxo1 siRNA treated cells when compared to negative control siRNA (NC siRNA) treated cells. The graph showed means ± SD (*n* = 2 each). **(E–H)** Inhibition of Foxo1 *in vitro* in splenocytes of miR-183^−/−^ **(E,F)** and miR-182^−/−^ **(G,H)** mice increased IFNγ, but not IL-6 production in response to anti-CD3/anti-CD28 or PMA/ionomycin stimulation. The cytokine level in Foxo1 siRNA treated samples were shown as the percentage of paired NC siRNA treated cells. The graphs were shown as means ± SD (*n* ≥ 4). Paired student t tests were performed (NC siRNA vs. Foxo1 siRNA); *, *p* < 0.05 and **, *p* < 0.01.

Previous studies have shown that miR-183C cluster miRNAs play an immune- and autoimmune-regulatory role through targeting Foxo1 directly (Stittrich et al., 2010; Ichiyama et al., 2016; Wang X. et al., 2018). We found an inverse relationship between the miR-183C miRNAs expression and Foxo1 protein expression in the splenocytes of MRL and MRL/lpr mice (Figure 7A). Consistent with the marked increase of miR-183C miRNAs, there was a significant decrease in the Foxo1 protein expression in MRL/lpr splenocytes. Knocking down miR-182 *in vitro* alone with antagomir-182 or miR-183C cluster with the mixes of antagomir-182, -96, -183 in MRL/lpr splenocytes increased Foxo1 protein expression (Figure 7B). Further, Foxo1 protein expression was significantly increased in CD4^+^ T cells from miR-182^−/−^B6/lpr and miR-183^−/−^B6/lpr compared to the CD4^+^ T cells from control mice (Figure 7C). Together, these data demonstrated that Foxo1 is the target of miR-183C miRNAs in splenocytes and T cells of MRL/lpr and B6/lpr mice. Deletion of miR-182 or miR-183C increased Foxo1 expression (Figure 7C) and reduced IFNγ in B6/lpr splenocytes in response to anti-CD3 plus anti-CD28 or PMA plus ionomycin stimulation (Figures 6A,D). To determine whether miR-183C regulates the production of cytokines in splenocytes through targeting Foxo1, we inhibited Foxo1 expression with Foxo1 siRNA *in vitro* in splenocytes from miR-183C^−/−^B6/lpr and miR-182^−/−^B6/lpr. We confirmed the significant reduction of Foxo1 mRNA expression level in the Foxo1 siRNA transfected cells (Figure 7D). In Foxo1 siRNA transfected cells, there was a significantly increased IFNγ production in response to anti-CD3 plus anti-CD28 or PMA plus ionomycin stimulation when compared to control siRNA transfected cells (Figures 7E,G). Suppression of Foxo1 *in vitro* has minimal effect on IL-6 production in activated splenocytes from miR-183C^−/−^B6/lpr and miR-182^−/−^B6/lpr mice (Figures 7F,H). This is consistent with the finding that deletion of miR-182 or miR-183C *in vivo* has minimal effect on IL-6 in *in vitro* activated splenocytes (Figures 6B,E). Together, our data suggested that miR-183C acts through Foxo1 to regulate the production of inflammatory cytokine IFNγ, a key pathogenic cytokine in the *lpr* lupus model.

## Discussion

Several studies have revealed that miR-183C miRNAs are critically involved in immunity and autoimmunity via regulating T cell activation, inflammatory cytokine IFNγ production, Tregs, and pathogenic Th17 cells differentiation and function (Stittrich et al., 2010; Kelada et al., 2013; Ichiyama et al., 2016; Wan et al., 2016; Thiel et al., 2019). Dysregulated miR-183C miRNAs expression has been identified in lupus and other human diseases (Dai et al., 2010; Chen J. Q. et al., 2017; Chen Y. J. et al., 2017; Liguori et al., 2018; Thiel et al., 2019). This study demonstrated that *in vitro* inhibition and *in vivo* deletion of miR-183C or miR-182 alone significantly reduces inflammatory cytokine IFNγ production in *in vitro* activated splenic lymphocytes from *lpr* lupus mice. This finding was significant as IFNγ is a key pathogenic cytokine in *lpr* lupus mice (Fan and Wüthrich, 1997; Peng et al., 1997). We further demonstrated that miR-183C and miR-182 regulate inflammatory IFNγ production in *in vitro* activated splenocytes *via* targeting Foxo1. Deletion of either miR-183C or miR-182 alone had no changes on the serum levels of IFNγ, IL-6, or other selected lupus-related cytokines, such as TNFα, IL-10, and IL-17 (Supplementary Figures S4A,B). Consistent with the previous data showing that miR-182 is largely dispensable for adaptive immune cell development, deletion of either miR-182 alone or miR-183C cluster did not affect lymphocyte composition in the spleens of B6/lpr mice. Importantly, we found that deletion of the miR-183C cluster significantly reduced serum anti-dsDNA level and IgG and C3 immunocomplex deposition in the kidneys of B6/lpr mice. Deletion of miR-182 alone suppressed immunocomplex IgG deposition in the B6/lpr kidneys but had only minor effects on the serum level of anti-dsDNA autoantibodies.

A previous study has shown that inhibition of miR-182 with antagomir-182 attenuated lupus nephritis in MRL/lpr mice via targeting Foxo1 (Wang X. et al., 2018). Our study revealed that deletion of miR-183C suppressed anti-dsDNA production and kidney IgG immunocomplex deposition in B6/lpr but had no obvious effect in renal function and histopathology (Figures 2, 4, and Table 2). This could be due to the observation that B6/lpr mice, unlike MRL/lpr, do not show significant clinical pathology and lupus nephritis development (Santiago-Raber et al., 2007). In contrast to the upregulated miR-183 in MRL/lpr splenocytes, a recent study showed that the expression of miR-183 was decreased in renal tissues of MRL/lpr mice and human patients with LN (Li et al., 2019). Intraperitoneal delivery of miR-183 mimics to MRL/lpr mice attenuated murine lupus nephritis with reduced anti-dsDNA antibodies, BUN through targeting mTOR (Li et al., 2019). These data suggested a tissue-specific dysregulation and function of miR-183 in lupus. The reason of why both administration of miR-183 mimic (Li et al., 2019) and deletion of miR-183-96-182 cluster have a suppressive effect on anti-dsDNA antibodies (Figure 2F) is unknown and perplexing. The previous studies have shown that deletion of miR-182 *in vivo* could not confirm the role of miR-182 on T cell-mediated immune responses that were determined by *in vitro* and *ex vivo* inhibition of miR-182 with antagomir (Stittrich et al., 2010; Pucella et al., 2015). It should be noted the off-target effect of systemic administration of antagomir and miRNA mimic *in vivo* is one possibility for the discrepancy of the data (Ichiyama and Dong, 2019).

miR-183C miRNAs have been shown to be involved in autoimmunity through promoting Tregs and Th17 cells differentiation and function. In our studies, we found that deletion of either miR-182 or miR-183C did not affect Treg development (Figures 5C,G). Also, compared to controls, there were no significant differences in the production of IL-17 in splenocytes from miR-182^−/−^B6/lpr and miR-183C^−/−^B6/lpr mice, which were stimulated with either non-pathogenic or pathogenic Th17 stimuli (Figures 6C,F). A previous study showed that the B6 mice with miR-182 deficiency had normal B cell development and antibody response to the T cell-dependent antigen NP-CGG (Pucella et al., 2015). However, Li et al. (2016) reported that miR-182 deficiency impaired early T cell-dependent immune response. The miR-182^−/−^ B6 mice had reduced production of antigen-specific IgM and IgG1 at early time point but not late time point after immunization with NP-CGG. Mechanistically, Li et al. (2016) showed that miR-182 deficiency impaired the antigen-specific antibody response by suppressing the generation of extra-follicular plasma cells without perturbing the expansion of T_FH_ and GCB cells. The authors further confirmed the critical role of miR-182 in extra-follicular B cell response by showing that the miR-182 knockout B6 mice were not able to respond to T-cell-independent type 2 antigen NP-Ficoll, which typically elicited an extrafollicular B-cell response (Li et al., 2016). Consistent with this report (Li et al., 2016), we found that deletion of miR-182 or miR-183C did not affect T_FH_, GCB and CD19^−^CD138^+^plasma cells (Figure 5). Further investigation of T cell-dependent and independent B cell responses is necessary to understand the mechanism underlying reduced serum levels of IgG/IgM in miR-182^−/−^ B6/lpr and anti-dsDNA in miR-183C^−/−^ B6/lpr mice.

Foxo1 is a well-defined target of miR-183C miRNAs in immune cells. In our studies, we confirmed that miR-182 and miR-183C miRNAs targeted Foxo1 in splenic lymphocytes of *lpr* lupus mice. Moreover, we demonstrated that inhibition of Foxo1 *in vitro* was able to reverse the suppressive effect of miR-183C and miR-182 deletion on IFNγ production in anti-CD3 plus anti-CD28 or PMA plus ionomycin activated splenocytes (Figures 7E,G). This suggested that miR-182 and miR-183C regulate IFNγ production in splenocytes *via* targeting Foxo1. Further studies to determine other specific target genes of miR-183C and miR-182, in addition to *Foxo1*, may help us to better understand the similar and divergent role of miR-182 and miR-183C in regulation of autoinflammatory response.

In conclusion, our data demonstrated that deletion of miR-183C reduced anti-dsDNA autoantibody production, IgG and C3 immunocomplex deposition in B6/lpr kidneys. Although the cellular and molecular mechanism of miR-183C regulating anti-dsDNA autoantibody production remains to be clarified, these data revealed a regulatory role of miR-183C on anti-dsDNA production in lupus. Furthermore, our data validated that Foxo1 is an important target gene of miR-183C miRNAs and miR-182, by which miR-183C and miR-182 regulate IFNγ production in splenocytes following antigen stimulation.

## Data Availability

The original contributions presented in the study are included in the article/Supplementary Material, further inquiries can be directed to the corresponding authors.
