# Supplementary material for: Deletion of microRNA-183-96-182 Cluster in Lymphocytes Suppresses Anti-DsDNA Autoantibody Production and IgG Deposition in the Kidneys in C57BL/6-Faslpr/lpr Mice
Source: Front Genet. 2022 Jul 7;13:840060. doi: 10.3389/fgene.2022.840060 (PMC9301314; doi:10.3389/fgene.2022.840060)
Supplement: Supplementary file 1 [file DataSheet1.PDF]

Supplementary Material

1 Supplementary Figures

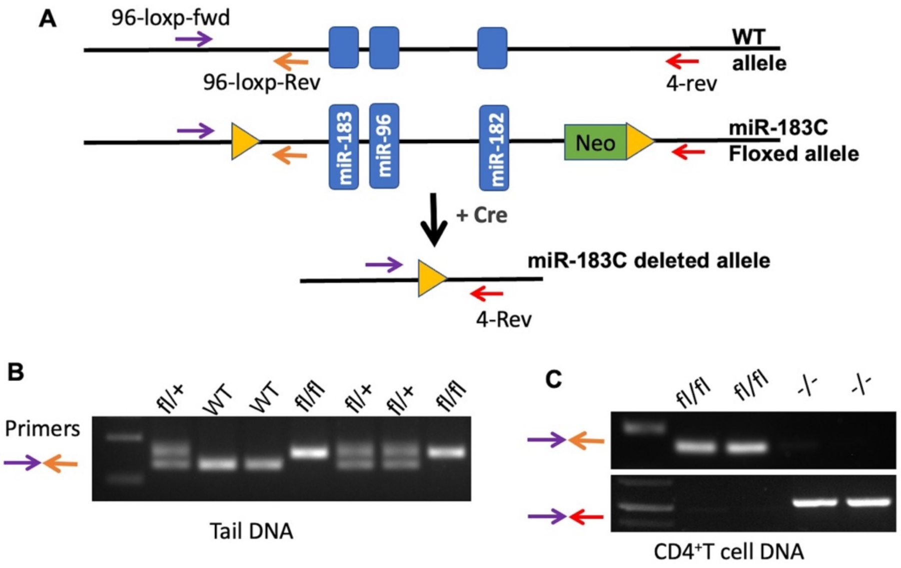

Supplemental Figure 1. Generation of conditional miR-183C knockout mice. (A) Schematic illustration of wild type (WT) allele, miR-183C floxed allele and recombined (deleted) allele. In the miR-183C floxed allele (miR-183C^{fl}), the whole miR-183-96-182 cluster was flanked by two loxP sequences (yellow triangle). Following Cre recombination, the sequence flanked by the two loxP sequences was deleted to generate the miR-183C deleted allele. The position of the primers (96-loxP-fwd, 96-loxP-Rev and 4-Rev) for genotyping WT, floxed (fl), and deleted allele are shown. **(B)** PCR genotyping of WT (+/+) and floxed allele (fl/+, fl/fl) with tail DNA from mice. The WT (+/+) mice showed a band at about 110bp; the homozygotes (fl/fl) showed a band at about 160bp; the heterozygotes (fl/+) showed both 110bp and 160bp size bands. **(C)** PCR genotyping to validate the depletion of miR-183C in CD4^{+} T lymphocytes of the conditional knockout miR-183C$^{-/-}$B6/lpr (-/-) mice, which are miR-183C$^{fl/fl}$B6/lpr with the expression of hCD2-iCre. With the primer set 96-loxP-fwd and 96-loxP-Rev, there was a band of about 160bp in CD4^{+} T cell DNA samples from the control miR-183C$^{fl/fl}$B6/lpr (fl/fl) mice, which do not carry hCD2-iCre. There was no band in the CD4^{+} T cell DNA samples from miR-183C$^{-/-}$B6/lpr (-/-) due to deletion of the binding site for the 96-loxP-Rev primer. With the primer set 96-loxP-fwd and 4-Rev, we detected a recombined band of about 500 bp size in the CD4^{+} T cell genomic DNA samples from miR-183C$^{-/-}$B6/lpr (-/-) mice, but not in the CD4^{+} T cell DNA samples from miR-183C$^{fl/fl}$B6/lpr (fl/fl), which have unrecombined allele and the distance between the primers is too long to be amplified. The genotyping primers' sequence are as follow. 96-loxP-fwd: 5'-CAG TTC TAG CAC CCA GTT TT-3'; 96-loxP-Rev: 5'-GCC AAA AGG CCA AGG AAG TC-3'; 4-Rev: 5'-AGG CCG CAG CTC CAG ATG AC-3'.

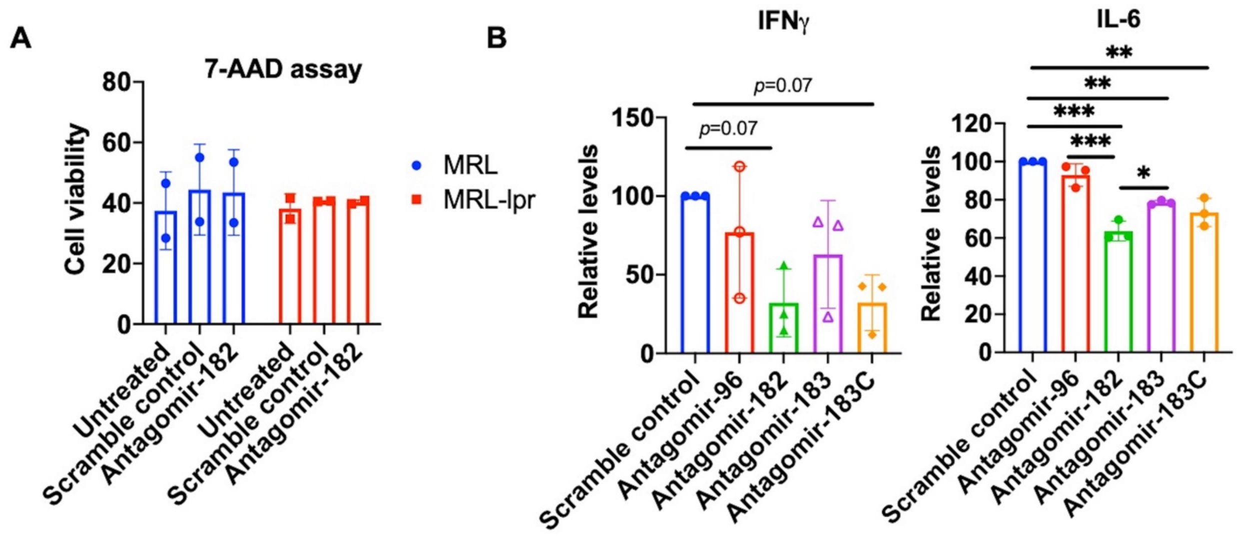

Supplemental Figure 2. *In vitro* study suggests that miR-182 plays the major role in regulating IFNγ and IL-6 production in activated splenocytes. (A) Antagomir treatment did not affect cell viability. The splenocytes from MRL-lpr mice were treated with or without antagomirs for 48h, and then stained with 7-amino-actinomycin D (7-AAD) for flow cytometry analysis of the cell viability. The graph showed means ± SD (n=2). **(B)** Inhibition of miR-182 alone has similar suppression effect on IFN*γ* and IL-6 production as inhibition of miR-183C whole cluster miRNAs. The splenocytes from MRL/lpr mice were treated with either individual antagomir for miR-96, -182, -183, or the mix of 3 antagomirs. Twenty-four hours after antagomir treatment, the cells were activated with LPS for 48hrs, and the cell culture supernatant were collected for ELISA. The IFNγ and IL-6 cytokine levels in the specific antagomir treated cells were showed as the percentage of paired scrambled control antagomir-treated cells. The graph showed means± SD (n=3 each). Paired student *t* tests were performed (scrambled control antagomir *vs* specific antagomir, specific antagomir *vs* the others); *, $p < 0.05$, **, $p < 0.01$, and ***, $p < 0.001$.

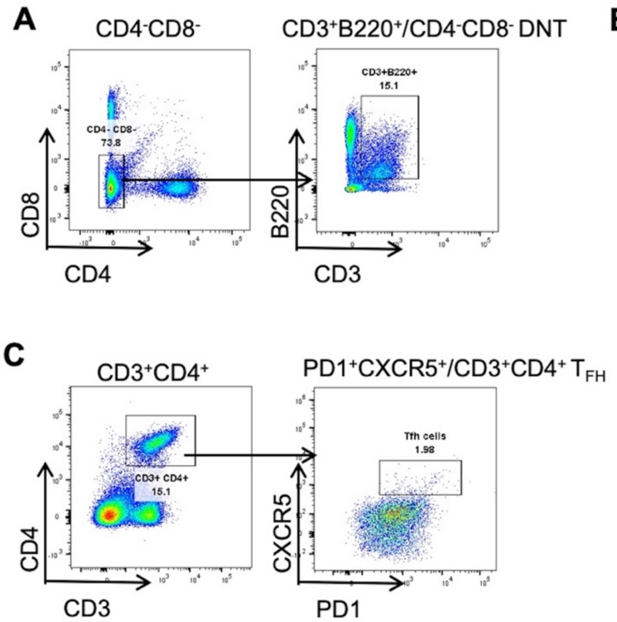

Supplemental Figure 3. Flow cytometry analysis of immune cell subsets population. (A) The gated CD4$^-$CD8$^-$ cells were further checked for the expression of CD3 and B220 to determine the population of CD4$^-$CD8$^-$ CD3$^+$B220$^+$ double negative T cells (DN T) in splenocytes. **(B)** The gated CD4$^+$ cells were further interrogated for the expression surface CD25 marker and intercellular Foxp3 protein to determine the population of CD4$^+$CD25$^+$Foxp3$^+$ Tregs. **(C)** The gated CD3$^+$CD4$^+$ were further interrogated for the expression of CXCR5 and PD-1 to determine the population of CD3$^+$CD4$^+$ CXCR5$^+$ PD-1$^+$ T_{FH} cells. **(D)** The gated CD19$^+$ cells were further checked for the expression of GL7 and IgD to determine CD19$^+$GL7$^+$IgD$^-$ GCB population. The population of CD19$^-$CD138$^+$ plasma cell population were gated out directly from splenocytes. Representative flow plots were shown for the gating of each specific cell population in splenocytes.

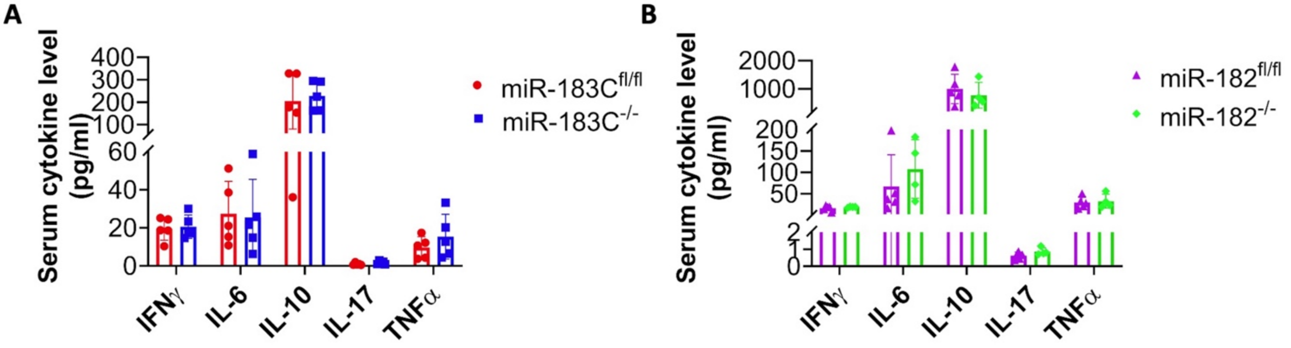

Supplemental Figure 4. Depletion of miR-183C or miR-182 has no obvious effect on serum cytokine levels. The levels of IFNγ, IL-6, IL-10, IL-17, and TNF-α in the serum from miR-183C$^{-/-}$ B6/lpr (miR-183C$^{-/-}$) **(A)**, miR-182$^{-/-}$B6/lpr (miR-182$^{-/-}$) **(B)** and their respective control miR-183C$^{fl/fl}$B6/lpr (miR-183C$^{fl/fl}$) and miR-182$^{fl/fl}$B6/lpr (miR-182$^{fl/fl}$) mice were quantified by Multiplex ELISA assay. The graphs showed means ± SD (n=5 each). Unpaired student *t* tests were performed (miR-183C$^{fl/fl}$ *vs* miR-183C$^{-/-}$, miR-182$^{fl/fl}$ *vs* miR-182$^{-/-}$). No statistical significance was observed.
